# Ocular Phenotype Associated with *DYRK1A* Variants

**DOI:** 10.3390/genes12020234

**Published:** 2021-02-05

**Authors:** Cécile Méjécase, Christopher M. Way, Nicholas Owen, Mariya Moosajee

**Affiliations:** 1 UCL Institute of Ophthalmology, London EC1V E9L, UK; smgxcme@ucl.ac.uk (C.M.); chrisw556@gmail.com (C.M.W.); n.owen@ucl.ac.uk (N.O.); 2 Moorfields Eye Hospital NHS Foundation Trust, London EC1V 2PD, UK; 3 Great Ormond Street Hospital for Children NHS Foundation Trust, London WC1N 3JH, UK; 4 The Francis Crick Institute, London NW1 1AT, UK

**Keywords:** *DYRK1A*, *DYRK1A*-related intellectual disability syndrome, mental retardation 7, ocular phenotype, optic nerve hypoplasia, strabismus and refractive error

## Abstract

Dual-specificity tyrosine phosphorylation-regulated kinase 1A or *DYRK1A,* contributes to central nervous system development in a dose-sensitive manner. Triallelic *DYRK1A* is implicated in the neuropathology of Down syndrome, whereas haploinsufficiency causes the rare *DYRK1A*-related intellectual disability syndrome (also known as mental retardation 7). It is characterised by intellectual disability, autism spectrum disorder and microcephaly with a typical facial gestalt. Preclinical studies elucidate a role for *DYRK1A* in eye development and case studies have reported associated ocular pathology. In this study families of the DYRK1A Syndrome International Association were asked to self-report any co-existing ocular abnormalities. Twenty-six patients responded but only 14 had molecular confirmation of a *DYRK1A* pathogenic variant. A further nineteen patients from the UK Genomics England 100,000 Genomes Project were identified and combined with 112 patients reported in the literature for further analysis. Ninety out of 145 patients (62.1%) with heterozygous *DYRK1A* variants revealed ocular features, these ranged from optic nerve hypoplasia (13%, 12/90), refractive error (35.6%, 32/90) and strabismus (21.1%, 19/90). Patients with *DYRK1A* variants should be referred to ophthalmology as part of their management care pathway to prevent amblyopia in children and reduce visual comorbidity, which may further impact on learning, behaviour, and quality of life.

## 1. Introduction

*DYRK1A* is composed of 13 exons, which encode the 763 amino acid dual-specificity tyrosine phosphorylation-regulated kinase 1A, or DYRK1A protein. This proline directed kinase is part of the DYRK family of five members (DYRK1A, DYRK1B, DYRK2, DYRK3 and DYRK4). It is highly expressed in the developing and adult central nervous system (CNS) [1,2]. Once activated by auto-phosphorylation [3], it phosphorylates serine or threonine residues of transcription, splicing, synaptic, apoptotic and translocation factors [4,5] to influence neurogenesis, neural differentiation, synaptic function and apoptotic pathways [5]. Within the CNS, DYRK1A is involved in dendritic arborization [6,7,8], cell cycle control, neural development and axon growth through interactions with various processes such as the nuclear factor of activated T-cells (NFAT) and cAMP response-element binding (CREB) pathways [6,9,10,11].

*DYRK1A* is located on chromosome 21q22.13 within the critical region causing Down syndrome (also known as Trisomy 21). Overexpression of *DYRK1A* produces similar neurodevelopmental [12,13,14] and neurodegenerative [15,16,17] changes to animal disease models with Down syndrome. Haploinsufficiency of *DYRK1A* through chromosomal loss of heterozygosity, microdeletions or intragenic mutation causes the rare *DYRK1A*-related intellectual disability syndrome, which was first detected through karyotype analysis of partial monosomy of chromosome 21 [18,19,20]. Comparative genomic hybridization has since allowed the discovery of a number of cases of chromosome 21 microdeletions, and the syndrome was termed autosomal dominant mental retardation 7 (MRD7, MIM#614104) [21,22,23,24,25,26]. Next-generation sequencing has allowed the identification of numerous point and frameshift variants in *DYRK1A* [27,28,29,30,31].

*DYRK1A*-related intellectual disability syndrome is characterised by a broad syndromic phenotype. It has a particular facial gestalt of deep-set eyes, short nose with a broad tip, up-slanting palpebral fissures, turned down corners of the mouth, dysplastic ears and retrognathia with a broad chin. Hand and foot abnormalities include long tapered fingers, small hands and feet, toe syndactyly and high arched feet [32]. These features may not be seen until adulthood [22]. Microcephaly and moderate intellectual deficit are observed in 80% of cases, with the remaining 20% suffering from mild intellectual deficit. Other findings include psychomotor delay, febrile seizures, anxiety, altered stress reactions [22], spinal and thoracic features (including pectus excavatum, kyphosis and scoliosis) [32], gastrointestinal features (including feeding difficulties and gastroesophageal reflux) [32], cardiac features (including ventricular septal defect, patent ductus arteriosus, aortic valve disease), as well as renal features (agenesis and renal cysts) [32].

Animal models of *DYRK1A* haploinsufficiency report structural ocular defects and visual impairment. The optic lobe of *mnb Drosophila* is disproportionately more reduced than other areas of the brain and is associated with poor functional visual pattern fixation compared to controls [33]. *Dyrk1a^+/−^* mice have 25% smaller eyes (microphthalmia), a thinner retina, fewer retinal ganglion cells and altered retinal functioning measured by electroretinography (ERG) [34]. Mice triallelic for *Dyrk1a* also show poor retino-cortical visual processing and this effect is eliminated when *DYRK1A* copy number is normalised [35], suggesting the role of *DYRK1A* in visual system development is dose sensitive.

Several individuals with *DYRK1A* variants have been described with a variety of ocular pathologies [27,30,32,36]. However, it is unclear whether these eye defects are associated with the syndrome or incidental findings. In addition, several of the published case series do not investigate ophthalmic features. In this study, *DYRK1A* families belonging to the DYRK1A Syndrome International Association (DSIA) were asked to self-report any co-existent ocular disease together with their genetic results. Further patients were identified through the UK Genomics England 100,000 Genomes Project and combined with a review of the literature with the aim to outline the ocular phenotype seen in patients with *DYRK1A* variants.

## 2. Materials and Methods

This study had relevant local and national research ethics committee approvals (Moorfields Eye Hospital NHS Foundation Trust and the Northwest London Research Ethics Committee) and adhered to the tenets of the Declaration of Helsinki. Patients and relatives gave written informed consent for genetic testing through either the Genetic Study of Inherited Eye Disease (REC reference 12/LO/0141) or Genomics England 100,000 Genomes project (REC reference 14/EE/1112).

After consultation with the DYRK1A Syndrome International Association (DSIA), patient families were contacted to request anonymised information about their clinical diagnosis including their genetic result and any recorded ophthalmic phenotype. Guardians of patients provided informed consent. Patients without a confirmed molecular diagnosis were excluded from the analysis of ocular phenotype.

Participants of the UK 100,000 Genomes Project underwent whole genome sequence (WGS) analysis [37]. High-throughput sequencing data were aligned to the human genome (GRCh38) using Isaac (Illumina Inc.), single nucleotide variants (SNVs) and indels (insertions and deletions) were identified, annotated and filtered using minor allele frequency in public datasets, predicted effect on protein and familial segregation (data release 11). Through the Genomics England data research embassy, variants were prioritised using the Intellectual Disability virtual gene panel (PanelApp, version 3.2), which includes *DYRK1A,* and variants identified as pathogenic or likely pathogenic were reported. Classification of such variants were based on the guidelines of the American College of Medical Genetics and Genomics (ACMG) [38]. Ocular features reported using human phenotype ontology (HPO) terms associated with the cases identified were analysed. All HPO terms observed are reported in Supplementary Table S1.

A review of the literature was also performed [19,20,22,23,24,25,26,27,28,29,30,32,39,40,41,42,43,44,45,46,47,48,49,50,51]. For each patient, their genetic defect and ocular phenotype was collected. Ocular features were categorised into refractive error, strabismus, enophthalmia (posterior displacement of the eye with sunken appearance), optic nerve abnormalities and other findings (Supplementary Table S2). Patients who unfortunately did not survive the neonatal period were not included in the analysis.

None of the recent publications reviewed in this study reported patients from the UK 100,000 Genome Project, which excluded any possible overlap between these cohorts. None of the patients from the DSIA self-reporting group reported being included in any other previously published studies.

## 3. Results

Twenty-six families were able to provide information on the proband’s ocular features, but only 14 had access to their genetic mutation (Table 1). Within this self-reporting patient cohort, the most common mutation type were deletions (*n* = 7), with chromosome 21 microdeletions in 4 patients and 3 small deletions. Four patients had in-frame nonsense mutations, two had frameshift single nucleotide duplications, and one patient had a missense mutation. The most common reported ocular feature was strabismus, reported in 100% (14/14) patients, of which exotropia was the most frequent in 8 of the 14 (57.1%). Intermittent exotropia secondary to a superior oblique palsy was reported in two patients (14.3%). Refractive error seen in 64.3% (9/14); two with confirmed hypermetropic astigmatism and one with hypermetropia. Optic nerve hypoplasia was seen in 42.9% (6/14) of patients. Other abnormalities included microphthalmia in 2/14 patients (14.3%) and corneal opacities in 2/14 patients (although one patient had also been diagnosed with Schnyder’s corneal dystrophy secondary to a concomitant mutation in *UBIAD1*). Iris coloboma, cataracts and congenital nasolacrimal duct obstruction were reported in individual patients.

Nineteen patients from the UK Genomics England 100,000 Genomes Project were identified with 18 unique heterozygous pathogenic or likely pathogenic variants in DYRK1A (Table 1 and Table S1) [37]. Seven patients had nonsense mutations (2 had c.613C>T, p.[Arg205*]), five missense variants, four splice site variants, two frameshift mutations and one in-frame deletion. Nine out of the 19 patients had ocular features (47.4%), the most common being optic nerve hypoplasia (*n* = 3, 15.8%) (Table 1 and Table S1). Non-specific HPO terms were provided in eight patients, such as “Abnormality of the eye” (HP:0000478) or “Visual impairment” (HP:0000504). This lack of clinical detail suggests that patients were not recruited into the study by ophthalmologists or lacked formal ophthalmic diagnosis.

There were 112 patients reported in the literature with either chromosome 21 heterozygosity, or specific disease-causing variants involving *DYRK1A* [19,20,22,23,24,25,26,27,28,29,30,32,39,40,41,42,43,44,45,46,47,48,49,50]. Chromosomal abnormalities were reported in 25 patients and included large deletions, translocations, inversions, inversion/deletions and complex defects. *DYRK1A*-related disease was mostly caused by frameshift variants (32/112), followed by nonsense (29/112), missense (16/112) and splice site (10/112) mutations. Ocular features were reported in 59.8% of cases (67/112), were declared absent in 15.2% (17/112), and not reported in 25% of cases (28/112). As with patients from the UK Genomics England 100,000 Genomes Project, numerous ocular phenotypes were ambiguous [19,20,22,23,24,25,26,27,28,29,30,32,39,40,41,42,43,44,45,46,47,48,49,50,51].

**Figure 1:**
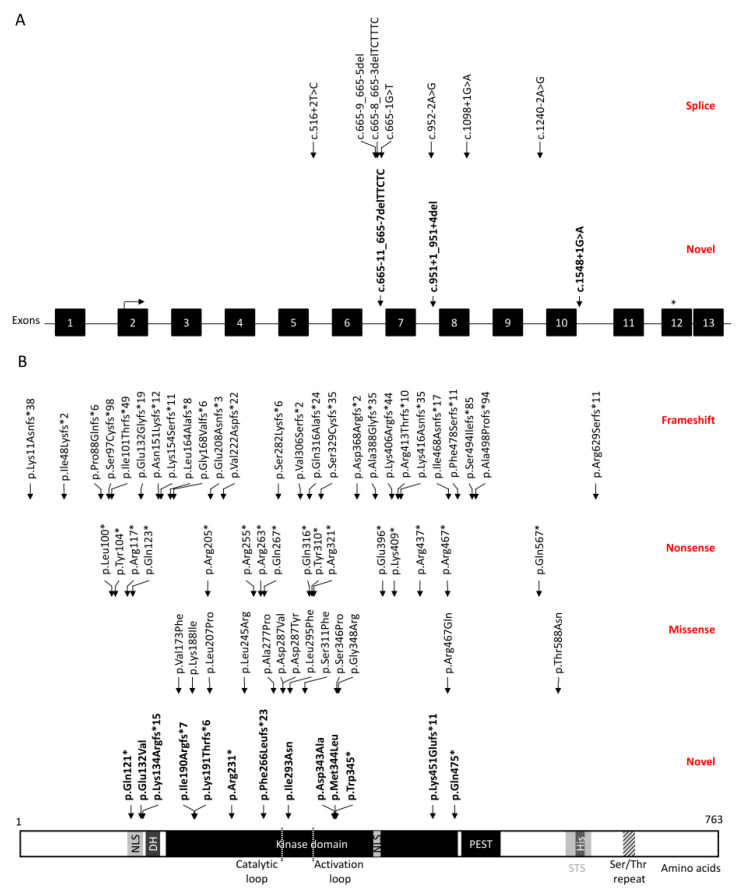
Mutational spectrum of *DYRK1A*-related intellectual disability syndrome. (**A**) Splice site variants are depicted across the 13 exons of *DYRK1A* (NM_001396.5). Novel splice site variants are in bold. (**B**) Amino acid change of frameshift, nonsense, missense and novel variants are mapped across the DYRK1A protein (NP_001387.2, Uniprot Q13627). Nuclear localization signals (NLS) between amino acid position 117–134 and position 386–394 are marked in grey; DYRK homology (DH) box [amino acid 137–153] is in grey; protein kinase domain [amino acid 159–479] is in black; catalytic loop [amino acid 285–287] and activation loop [amino acid 319–321] are depicted in hatched black box; PEST domain [amino acid 482–525] is in black; speckle-targeting signal (STS) [amino acid 596–624] is in grey; histidine repeat (His) [amino acid 607–619] in dark grey; and a Serine/Threonine (Ser/Thr) repeat [amino acid 659–672] is in hatched black box [3,52]. The in-frame deletion, c.914_919del p.(Ile305_Asp307delinsAsn), is not reported in this figure.

The 112 reported patients were added to our self-reporting cohort of 14 patients and the 19 patients from the 100,000 Genomes Project to generate a total of 145 patients from 144 unrelated families with 108 unique disease-causing variants involving *DYRK1A* (Figure 1). Chromosomal abnormalities accounted for 25% of these (27/108). Among the 75% of single nucleotide variants (81/108); 32 frameshift variants (39.5%) were reported in 39 patients, 20 nonsense variants (24.7%) in 40 patients, 17 missense variants (33.3%) in 22 patients and 10 splice site variants (12.4%) in 14 patients. This cohort identified 17 novel variants (Figure 1); five frameshift variants (c.398del p.(Lys134Argfs*15), c.569_572del p.(Ile190Argfs*7), c.572_575del p.(Lys191Thrfs*6), c.796delT p.(Phe266Leufs*23), c.1350dup p.(Lys451Glufs*11)), four nonsense variants (c.361C>T p.(Gln121*), c.691C>T p.(Arg321*), c.1035G>A p.(Trp345*), c.1423C>T p.(Gln475*)), four missense variants (c.395A>T p.(Glu132Val), c.1028A>C p.(Asp343Ala), c.1030A>T p.(Met344Leu), c.878T>A p.(Ile293Asn)), three splice site variants (c.665-11_665-7delTTCTC, c.951+1_951+4del, c.1548+1G>A) and one in frame deletion (c.914_919del p.(Ile305_Asp307delinsAsn)).

**Figure 2:**
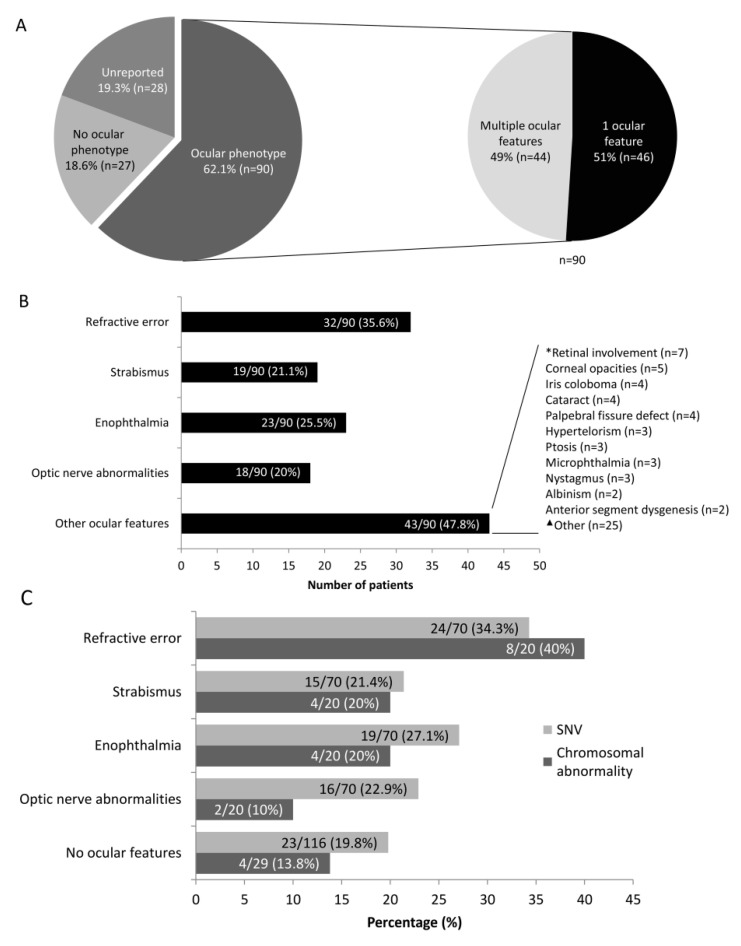
Ocular features reported in patients with *DYRK1A*-related intellectual disability syndrome and genetic diagnosis. (**A**) Ninety patients are reported with one or multiple ocular features. (**B**) Ocular features were divided into five categories; refractive error (including hyperopia/hypermetropia, myopia, astigmatism) was observed in 32 patients, strabismus (including exotropia, exophoria, esotropia, pseudo-exotropia) in 19 patients, enophthalmia (including sunken eye appearance and deep-set eyes) in 23 patients, optic nerve abnormalities in 18 patients and other ocular features in 43 patients. Within the other ocular features group * retinal involvement was seen in 7 patients including retinal detachment (*n* = 2, occurred at 4 months of age in a male patient with bilateral microphthalmia, and in a second female patient age 59 years with no other clinical details), retinal dystrophy (*n* = 2), abnormal fundus findings (*n* = 3). ^▲^ Other included non-specific terms such as visual impairment (*n* = 7) and abnormality of the eye (*n* = 4), cortical visual impairment, photosensitivity, congenital nasolacrimal duct obstruction (*n* = 2), blepharophimosis, sclerocornea. (**C**) Comparison of ocular features between patients with *DYRK1A* SNV and chromosomal abnormalities across the 4 main categories, including refractive error, strabismus, enophthalmia and optic nerve abnormalities. In addition, we highlight the number of families that had no reported ocular features amongst the two groups. Overall, no statistical difference was seen between the two mutation groups using chi squared test.

Ocular features were seen in 62.1% (90/145) of patients with *DYRK1A* variants including SNVs and chromosomal aberrations. None were seen in 18.6% (27/145), and information was unavailable in the remaining 19.3% (28/145) (Figure 2A and Supplementary Table S2). Patients reported one ocular feature in 51% (46/90) and multiple ocular features in 49% (44/90) (Figure 2A). The most common pathologies seen were refractive error (including hyperopia/hypermetropia, myopia, astigmatism) seen in 35.6% of individuals (32/90) (Figure 2B). Strabismus (including exotropia, exophoria, esotropia) was seen in 21.1% of patients (19/90). Enophthalmia (including deep-set eyes) were reported in 23 individuals (25.6%) (Figure 2B). Optic nerve abnormalities were seen in 20% patients (*n* = 18 including optic nerve hypoplasia in 12, optic disc pallor, optic nerve atrophy, small/thin optic nerve, chiasma dysfunction) (Figure 2B). Forty-three patients displayed other associated ocular features outlined in Figure 2B.

Patients with *DYRK1A* SNV only (*n* = 116) and chromosomal aberrations (*n* = 29) were divided to assess if there was any difference in ocular features between the two groups (Figure 2C). Seventy out of 116 patients (60.3%) with SNVs had ocular features, and no eye abnormalities were seen in 19.8% (23/116); information was unavailable in the remaining 19.8% (23/116) (Supplementary Table S2). Twenty out of 29 patients (69%) with chromosomal aberrations had ocular features, and no eye abnormalities were seen in 13.8% (4/29); information was unavailable in the remaining 17.2% (5/29) (Supplementary Table S2). There was no significant difference between the ocular features seen in the two groups. Patients reported one ocular feature in 62.9% (44/70) of the SNV group compared to 65% (13/20) in the chromosomal group. Multiple ocular features in 37.1% (26/70) and 35% (7/20) in the SNV and chromosomal aberration group, respectively. The most common pathologies seen were refractive error seen in 34.3% (24/70) and 40% (8/29) in the SNV and chromosomal aberration group, respectively (Figure 2C).

## 4. Discussion

*DYRK1A*-related intellectual disability syndrome is a rare disease. An accurate estimation of the incidence compared to the general population is yet to be established. Patients display several pathognomonic and associated clinical features and are likely monitored by a large multidisciplinary care team, hence gathering information on the complete phenotype can be challenging. The DSIA provided a useful platform to collect genetic and clinical data from several motivated patient guardians from across the world. This cohort, combined with the 100,000 Genomes Project and the literature, generated 145 patients with *DYRK1A*-related disease. At least 62% of these patients (90/145) displayed ocular manifestations. This will guide future management, including early ophthalmology review, and inform clinicians of which features may present. This is an under-reported association with missing data in 19.3% of cases, and non-specific terms relating to ocular/visual abnormalities recorded. Hence, more detailed phenotyping is required, and prospective epidemiological studies would help determine the actual incidence of these ocular features in comparison to the general population.

One hundred and eight *DYRK1A* variants have been reported to cause *DYRK1A*-related intellectual disability syndrome, 75% are single nucleotide variants (81/108), with 17 being novel from this study. The most prevalent mutations were loss-of-function nonsense (40/108) and frameshift (39/108). Chromosomal rearrangements involving *DYRK1A* account for 25% of this cohort with 68.9% (20/29) of this subset displaying an ocular phenotype. Although, observed ocular features between patient groups with and without chromosomal abnormalities were similar, these rearrangements affect several genes which may contribute to extra-*DYRK1A*-related features. Particular single nucleotide variants, which were more commonly seen amongst patients, showed a variable ocular phenotype. For example, the nonsense variant c.613C>T p.(Arg205*) was reported in 8 unrelated patients, of which six reported single or multiple eye defects including refractive error, exotropia and ptosis; information was unavailable for the 2 remaining patients. However, in two unrelated patients with the c.691C>T p.(Arg231*) nonsense variant, each displayed an iris coloboma [51]; and in two patients with the missense variant c.860A>T p.(Asp287Val), both developed cataracts, one self-reported in our cohort, the other was childhood bilateral cataracts (examination at 6.6 years-old) [51]. A larger cohort of patients with similar variants would be needed to confirm any phenotype–genotype correlation. The frameshift c.143_144delTA p.(Ile48Lysfs*2) and missense c.1036T>C p.(Ser346Pro) variants were reported in two patients each, with either an unremarkable ocular examination or eye findings including hypermetropia or exotropia, respectively. These phenotypic differences in those with the same *DYRK1A* variant may be explained by potential variants in other genes, encoding genetic modifiers or non-coding regulatory elements, which affect its interactions [53]. Importantly, where the examination concludes an absence of ocular features, if detailed findings are not included in the literature, then it remains questionable whether all features have been adequately investigated.

Eighteen patients with genetic data were diagnosed with optic nerve abnormalities: 12/18 with optic nerve hypoplasia and 2/18 with optic atrophy. The associated variants were heterogeneous including two large deletions of chromosome 21, seven frameshift, five nonsense, two missense, one in-frame deletion, and one splice site variant. DYRK1A protein has an established role in optic nerve development, with haploinsufficient *Dyrk1a^+/-^* mice displaying a 40% smaller retinal ganglion cell layer and an optic nerve with 50% fewer axons than controls [34]. This is consistent with the association between *DYRK1A* haploinsufficiency and global cerebral hypodevelopment [27]. As part of the CNS, optic nerve development depends on the controlled regulation of apoptosis [54], and mechanisms that govern this include the caspase system [55,56,57]. Through phosphorylation of caspase 9 at amino acid position Thr125, DYRK1A prevents execution of the caspase 9-mediated intrinsic apoptotic pathway in the retina [34]. This results in impaired protection from apoptosis and may be a contributing mechanism to optic nerve hypoplasia in *Dyrk1a^+/-^* mice. In contrast, mice triallelic for *Dyrk1a* have increased ganglion and optic nerve fibre layer cellularity [35], perhaps because of increased protection against caspase 9-mediated apoptosis from excess DYRK1A. It would also explain some of the altered morphology in Down syndrome (with increased *DYRK1A* gene dosage) characterised by thicker retinas and altered visuo-cortical processing as observed by visual evoked potential (VEP) testing [58,59]. Further research into this pathophysiology will improve our understanding of the neuropathology of optic nerve hypoplasia in patients with *DYRK1A*-related intellectual disability syndrome [34].

Visual impairment is often seen in those with intellectual disability [60] and particularly Down syndrome [61]. In this gathered cohort of *DYRK1A*-related intellectual disability syndrome, 32 patients described refractive error. This is a common feature of syndromes related to developmental delay such as Down syndrome [58,62,63,64,65,66]. The percentage prevalence in this cohort (22.4%) is roughly twice that of the general population risk of refractive error, which is 11.7% [67]. Another common feature of Down syndrome is strabismus, where there is a five-fold increased risk compared to the population [64,65,68]. This cohort incidence of strabismus was 13.1% (19/145), 6.7 times larger than the global incidence of strabismus, estimated at 1.93% [69]. From this gathered cohort it is suggested that patients with *DYRK1A*-related intellectual disability syndrome are at an increased risk of developing both these features. However, more robust epidemiological studies are required to confirm this as this cohort is partly gathered from those under the management of a hospital eye service. The appropriate management of refractive error and strabismus, both of which are treatable causes of visual impairment, in patients with intellectual disability is complicated by difficulties in communication, attention and behavioural issues, which may reduce spectacle compliance [60,70]. However, the treatment of any preventable vision loss may reduce the social and behavioural difficulties seen in these patients.

Interestingly, it is estimated that 0.1–0.5% of patients with an ASD may have a mutation involving *DYRK1A* [30,71]. Between 40 and 88% of patients with molecularly confirmed *DYRK1A*-related intellectual disability syndrome are initially misdiagnosed with ASD, due to its overlapping clinical features and it being more common [27,28,30]. Hence, careful early phenotyping of patients or subsequent genotyping, may help prevent mis- or delayed diagnoses.

## 5. Conclusions

This study highlights that patients with *DYRK1A*-related intellectual disability syndrome may be at an increased risk of developmental ocular pathology compared to the general population, particularly optic nerve hypoplasia, refractive error and strabismus. Visual impairment further compounds the social, behavioural and emotional difficulties experienced by these patients and their families. Every patient with pathogenic *DYRK1A*-variants must undergo regular detailed ophthalmological assessment, especially in childhood, as part of their holistic care to minimise reversible visual loss and prevent amblyopia. It is recommended that as soon as a molecular diagnosis is confirmed, the patient is referred to ophthalmology if the patient has not already been reviewed by them. Depending on the findings i.e., refractive error and/or strabismus, follow-up will vary depending on the age and visual acuity of the child. However, once the patient is stable and past the age of developing amblyopia (7–8 years), they may be followed up annually by their local optometrist (into adulthood) or if significant limitations in functioning capability, in a multidisciplinary special education needs clinic. This study exemplifies the need to use standardised and precise phenotype vocabulary such as HPO terms, this will facilitate the accurate documentation and sharing of clinical features amongst health care professionals and permit further investigation of genotype-phenotype correlations. By increasing the awareness of the ocular associations of *DYRK1A*-related intellectual disability syndrome, a consensus on disease associations can be established, leading to more research into pathological mechanisms.

## Figures and Tables

**Table 1:** Ocular findings in patients with *DYRK1A*-related intellectual disability syndrome, from online patient group and from the UK Genomics England Ltd. 100,000 Genomes Project [37]. Fourteen patients and their guardians provided the full request of ocular diagnoses and mutation analysis. Nine patients with *DYRK1A*-related intellectual disability syndrome from the UK Genomics England Ltd. 100,000 Genomes Project experiences ocular features [37]. Human phenotype ontology (HPO) terms are used to describe phenotypes.

***DYRK1A* Families Belonging to the DYRK1A Syndrome International Association (DSIA)**					
**Patient ID**	***DYRK1A*** **Defect**	**Refractive** **Error**	**Strabismus**	**Optic Nerve Abnormalities**	**Other Findings**
1	c.569_572delTAAA p.(Ile190Argfs*7)	Amblyopic hyperopia			Congenital nasolacrimal duct obstruction
2	c.572_575del p.(Lys191Thrfs*6) ^1^		Exotropia		
3	c.613C>T p.(Arg205*)	Hyperopic astigmatism	Superior oblique palsy Intermittent exotropia		
4	c.691C>T p.(Arg231*)	Myopia			Iris coloboma (with CHARGE association)
5	c.763C>T p.(Arg255*)	Hypermetropia	Exotropia	Optic nerve hypoplasia	
6	c.860A>T p.(Asp287Val)	Hyperopia		Optic nerve hypoplasia	Bilateral cataracts
7	c.1035G>A p.(Trp345*) ^1^	Hyperopic astigmatism			
8	c.1217_1220del p.(Lys406Argfs*44)			Optic nerve hypoplasia	Schnyder corneal dystrophy (additional mutation to *UBIAD1*)
9	c.1350dupG p.(Lys451Glufs*11) ^1^	Myopic astigmatism	Esotropia		
10	c.1400dupG p.(Ile468Asnfs*17) ^1^	Refractive amblyopia	Superior oblique palsyIntermittent exotropia	Optic nerve hypoplasia	
11	del(21)(q22.12q23.3)				Microphthalmia Anterior segment dysgenesis Corneal opacities
12	del(21)(q22.13)			Optic nerve hypoplasia	Fundal pallor
13	del(21)(q22.13)	Myopic astigmatism	Esotropia	Optic nerve hypoplasia	
14	del(21)(q22.13q22.3)				Microphthalmia Sclerocornea
***DYRK1A*** **patients from the UK Genomics England 100,000 Genomes Project with ocular features**					
**Patient ID**	***DYRK1A*** **Defect**	**Refractive** **Error**	**Strabismus**	**Optic Nerve Abnormalities**	**Other Findings**
1	c.361C>T p.(Gln121*) ^1^				Severe visual impairment (HP:0001141)
2	c.613C>T p.(Arg205*)				Abnormality of the eye (HP:0000478)
3	c.763C>T p.(Arg255*)				Abnormality of the eye (HP:0000478)
4	c.878T>A p.(Ile293Asn) ^1^			Aplasia/ Hypoplasia of the optic nerve (HP:0008058)	Abnormality of the eye (HP:0000478)
5	c.914_919del p.(Ile305_Asp307delinsAsn) ^1^			Optic nerve hypoplasia (HP:0000609)	Abnormality of the eye (HP:0000478) Cataract (HP:0000518)
6	c.691C>T p.(Arg321*) ^1^				Anterior segment dysgenesis (HP:0007700)
7	c.1028A>C p.(Asp343Ala) ^1^				Hypertelorism (HP:0000316) Nonprogressive visual loss (HP:0200068)
8	c.1030A>T p.(Met344Leu) ^1^				Hypertelorism (HP:0000316) Nonprogressive visual loss (HP:0200068)
9	c.1548+1G>A ^1^			Optic nerve hypoplasia (HP:0000609)	Downslanted palpebral fissures (HP:0000494) Nystagmus (HP:0000639) Visual impairment (HP:0000505)

^1^ Variants previously not reported.

## Data Availability

The data presented in this study are available in Table S2.

## Supplementary Materials

The following are available online at https://www.mdpi.com/2073-4425/12/2/234/s1, Table S1: Nineteen patients from Genomics England Ltd. 100,000 genomes project experiences several features of *DYRK1A*-related intellectual disability syndrome [37]. Ocular features are in bold. HPO terms are used to describe phenotypes. Table S2: Review of ocular findings in patients with *DYRK1A*-related intellectual disability syndrome previously reported in the literature [19,20,22,23,24,25,26,27,28,29,30,32,39,40,41,42,43,44,45,46,47,48,49,50,51]. Not available: na. (excel file). Individual in red died prematurely.
