# Supplementary material for: Ocular Phenotype Associated with DYRK1A Variants
Source: Genes (Basel). 2021 Feb 5;12(2):234. doi: 10.3390/genes12020234 (PMC7915179; doi:10.3390/genes12020234)
Supplement: Supplementary file 1 [file genes-12-00234-s001.zip › DYRK1A supplementary data/Supplementary table S1 18-01-2021.docx]

**Supplementary table 1: Nineteen patients from the UK Genomics England Ltd 100,000 Genomes Project experiences several features of DYRK1A syndrome** [1]**.** Each Ocular features are in bold. HPO terms are used to describe phenotypes.

| Genotype | Phenotype |
| --- | --- |
| c.349C>T  p.(Arg117*) | Chronic constipation (HP:0012450) Delayed fine motor development (HP:0010862) Delayed gross motor development (HP:0002194) Delayed speech and language development (HP:0000750) Global developmental delay (HP:0001263) Hyperpigmentation of the skin (HP:0000953) Hypopigmentation of the skin (HP:0001010) Intellectual disability (HP:0001249) Limited elbow extension (HP:0001377) Limited knee flexion/extension (HP:0005085) Long phalanx of finger (HP:0006155) Microcephaly (HP:0000252) Morphological abnormality of the central nervous system (HP:0002011) Multifocal cerebral white matter abnormalities (HP:0007052) Seizures (HP:0001250) |
| c.361C>T  p.(Gln121*) | Abnormality of the palmar creases (HP:0010490)  Absent testis (HP:0010469) Atypical absence seizures (HP:0007270) Cerebral white matter hypoplasia (HP:0012430) Complex febrile seizures (HP:0011172) Dilation of lateral ventricles (HP:0006956) Edema of the dorsum of feet (HP:0012098) Generalized tonic-clonic seizures without focal onset (HP:0025190) Penoscrotal hypospadias (HP:0000808) Postnatal microcephaly (HP:0005484) **Severe visual impairment (HP:0001141)** Ventriculomegaly (HP:0002119) |
| c.395A>T  p.(Glu132Val) | Delayed CNS myelination (HP:0002188) Delayed fine motor development (HP:0010862) Delayed gross motor development (HP:0002194) Failure to thrive (HP:0001508) Global developmental delay (HP:0001263) Inability to walk (HP:0001508) Intellectual disability (HP:0001249) Microcephaly (HP:0000252) Proportionate short stature (HP:0003508) Small for gestational age (HP:0001518) |
| c.398del  p.(Lys134Argfs*15) | Autistic behavior (HP:0000729) Broad eyebrow (HP:0011229) Delayed fine motor development (HP:0010862)  Delayed gross motor development (HP:0002194) Delayed speech and language development (HP:0000750) Failure to thrive (HP:0001508) Global developmental delay (HP:0001263) Hypermelanotic macule (HP:0001034) Intellectual disability, severe (HP:0010864) Low posterior hairline (HP:0002162) Microcephaly (HP:0000252) Skin tags (HP:0010609) Small for gestational age (HP:0001518) |
| c.516+2T>C | Delayed speech and language development (HP:0000750) Febrile seizures (HP:0002373) Microcephaly (HP:0000252) Small for gestational age (HP:0001518) |
| c.613C>T  p.(Arg205*) | Delayed speech and language development (HP:0000750) Seizures (HP:0001250) Delayed fine motor development (HP:0010862) Intellectual disability (HP:0001249) Myoclonus (HP:0001336) Delayed gross motor development (HP:0002194) Microcephaly (HP:0000252) Global developmental delay (HP:0001263) Autistic behaviour (HP:0000729) |
| c.613C>T  p.(Arg205*) | Delayed speech and language development (HP:0000750) Delayed fine motor development (HP:0010862) Intellectual disability (HP:0001249) Abnormality of the outer ear (HP:0000356) Delayed gross motor development (HP:0002194) Abnormality of prenatal development or birth (HP:0001197) Microcephaly (HP:0000252) **Abnormality of the eye (HP:0000478)** Global developmental delay (HP:0001263) Morphological abnormality of the central nervous system (HP:0002011) Abnormal facial shape (HP:0001999) |
| c.665-11_665-7delTTCTC | Anteriorly placed anus (HP:0001545) Asymmetric growth (HP:0100555) Ataxia (HP:0001251) Delayed gross motor development (HP:0002194)  Delayed speech and language development (HP:0000750) Failure to thrive (HP:0001508) Global developmental delay (HP:0001263) Intellectual disability (HP:0001249) Microcephaly (HP:0000252) Proportionate short stature (HP:0003508) Seizures (HP:0001250) Small for gestational age (HP:0001518) Umbilical hernia (HP:0001537) |
| c.763C>T  p.(Arg255*) | **Abnormality of the eye (HP:0000478)** Delayed fine motor development (HP:0010862) Delayed gross motor development (HP:0002194) Delayed speech and language development (HP:0000750) Generalized hypotonia (HP:0001290) Global developmental delay (HP:0001263) Inability to walk (HP:0002540) Intellectual disability (HP:0001249) Microcephaly (HP:0000252) Scoliosis (HP:0002650) Seizures (HP:0001250) |
| c.796delT  p.(Phe266Leufs*23) | Abnormal facial shape (HP:0001999) Abnormality of the thenar eminence (HP:0001227) Ataxia (HP:0001251) Brisk reflexes (HP:0001348) Broad-based gait (HP:0002136) Cerebellar hemisphere hypoplasia (HP:0100307) Cerebellar hypoplasia (HP:0001321) Cerebellar vermis hypoplasia (HP:0001320) Cerebral palsy (HP:0100021) Clonus (HP:0002169) Delayed fine motor development (HP:0010862) Delayed gross motor development (HP:0002194) Delayed speech and language development (HP:0000750) Febrile seizures (HP:0002373) Global developmental delay (HP:0001263) Intellectual disability (HP:0001249) Long fingers (HP:0100807) Microcephaly (HP:0000252) Micrognathia (HP:0000347) Peripheral neuropathy (HP:0009830) Pes cavus (HP:0001761) Retrognathia (HP:0000278) Sensorimotor neuropathy (HP:0007141) Sensory neuropathy (HP:0000763) |
| c.878T>A  p.(Ile293Asn) | Abnormal facial shape (HP:0001999) Abnormality of male external genitalia (HP:0000032) **Abnormality of the eye (HP:0000478)** Abnormality of the outer ear (HP:0000356) **Aplasia/Hypoplasia of the optic nerve (HP:0008058)** Autistic behavior (HP:0000729) Bilateral cryptorchidism (HP:0008689) Delayed fine motor development (HP:0010862) Delayed gross motor development (HP:0002194) Delayed speech and language development (HP:0000750) Global developmental delay (HP:0001263) Hemifacial atrophy (HP:0011331) Intellectual disability (HP:0001249) Microcephaly (HP:0000252) Proportionate short stature (HP:0003508) Seizures (HP:0001250) Small for gestational age (HP:0001518) |
| c.883C>T  p.(Leu295Phe) | Abnormality of cardiovascular system morphology (HP:0030680) Abnormality of finger (HP:0001167)  Abnormality of the gastrointestinal tract (HP:0011024) Global developmental delay (HP:0001263) Intellectual disability (HP:0001249)  Microcephaly (HP:0000252)  Proportionate short stature (HP:0003508) |
| c.914_919del  p.(Ile305_Asp307delinsAsn) | Abnormal facial shape (HP:0001999) Abnormality of cardiovascular system morphology (HP:0030680) Abnormality of prenatal development or birth (HP:0001197) **Abnormality of the eye (HP:0000478)** Abnormality of the gastrointestinal tract (HP:0011024) Abnormality of the outer ear (HP:0000356) Abnormality of the palmar creases (HP:0010490) **Cataract (HP:0000518)** Cerebellar hypoplasia (HP:0001321) Cerebellar vermis hypoplasia (HP:0001320) Congenital microcephaly (HP:0011451) Failure to thrive (HP:0001508) Hypertonia (HP:0001276) Intellectual disability (HP:0001249) Intellectual disability, moderate (HP:0002342) Intrauterine growth retardation (HP:0001511) Inverted nipples (HP:0003186) Microcephaly (HP:0000252) Morphological abnormality of the central nervous system (HP:0002011) Neonatal hypotonia (HP:0001319) **Optic nerve hypoplasia (HP:0000609)** Poor suck (HP:0002033) Postnatal microcephaly (HP:0005484) Progressive microcephaly (HP:0000253) Proportionate short stature (HP:0003508) Pulmonary artery stenosis (HP:0004415) Short stature (HP:0004322) Single umbilical artery (HP:0001195) Small for gestational age (HP:0001518) Spasticity (HP:0001257) Underfolded helix (HP:0008577) |
| c.951+1_951+4del | Absent speech (HP:0001344)  Anal fistula (HP:0010447) Anteriorly placed anus (HP:0001545) Delayed fine motor development (HP:0010862) Delayed gross motor development (HP:0002194) Febrile seizures (HP:0002373) Global developmental delay (HP:0001263) Intellectual disability, severe (HP:0010864) Microcephaly (HP:0000252) Sparse scalp hair (HP:0002209) Trigonocephaly (HP:0000243) |
| c.691C>T  p.(Arg321*) | **Anterior segment dysgenesis (HP:0007700)** Delayed speech and language development (HP:0000750) Short stature (HP:0004322) |
| c.1028A>C  p.(Asp343Ala) | Delayed fine motor development (HP:0010862) Delayed gross motor development (HP:0002194) Global developmental delay (HP:0001263) **Hypertelorism (HP:0000316)** Intellectual disability (HP:0001249) Microcephaly (HP:0000252) **Nonprogressive visual loss (HP:0200068)** Proportionate short stature (HP:0003508) Spasticity (HP:0001257) |
| c.1030A>T  p.(Met344Leu) | Delayed fine motor development (HP:0010862) Delayed gross motor development (HP:0002194) Global developmental delay (HP:0001263) **Hypertelorism (HP:0000316)** Intellectual disability (HP:0001249) Microcephaly (HP:0000252) **Nonprogressive visual loss (HP:0200068)** Proportionate short stature (HP:0003508) Spasticity (HP:0001257) |
| c.1423C>T  p.(Gln475*) | Autistic behaviour (HP:0000729)  Contracture of the proximal interphalangeal joint of the 4th finger (HP:0009276) Delayed speech and language development (HP:0000750) Intellectual disability (HP:0001249) Microcephaly (HP:0000252)  Proportionate short stature (HP:0003508) |
| c.1548+1G>A | Coarse facial features (HP:0000280)  Delayed fine motor development (HP:0010862) Delayed gross motor development (HP:0002194) Delayed speech and language development (HP:0000750) **Downslanted palpebral fissures (HP:0000494)** Feeding difficulties in infancy (HP:0008872) Global developmental delay (HP:0001263) Hearing impairment (HP:0000365) Muscular hypotonia (HP:0001252) **Nystagmus (HP:0000639) Optic nerve hypoplasia (HP:0000609)** Protruding tongue (HP:0010808) **Visual impairment (HP:0000505)** Wide nasal bridge (HP:0000431) |
